# HDL Subclasses and the Distribution of Paraoxonase-1 Activity in Patients with ST-Segment Elevation Acute Myocardial Infarction

**DOI:** 10.3390/ijms24119384

**Published:** 2023-05-27

**Authors:** Saska Djekic, Jelena Vekic, Aleksandra Zeljkovic, Jelena Kotur-Stevuljevic, Srdjan Kafedzic, Marija Zdravkovic, Ivan Ilic, Sasa Hinic, Milivoje Cerovic, Milica Stefanovic, Marija Mihajlovic, Aleksandar Neskovic, Natasa Bogavac-Stanojevic

**Affiliations:** 1 Department of Laboratory Diagnostics Public Health Institution “Health Center”, 74000 Doboj, Bosnia and Herzegovina; saska.djekic@gmail.com; 2 Department of Medical Biochemistry, Faculty of Pharmacy, University of Belgrade, 11000 Belgrade, Serbia; aleksandra.zeljkovic@pharmacy.bg.ac.rs (A.Z.); jkotur@pharmacy.bg.ac.rs (J.K.-S.); natasa.bogavac@pharmacy.bg.ac.rs (N.B.-S.); 3 Clinical Hospital Center “Zemun”, 11000 Belgrade, Serbia; 4 Faculty of Medicine, University of Belgrade, 11000 Belgrade, Serbia; 5 Clinical Hospital Center “Bezanijska Kosa”, 11000 Belgrade, Serbia

**Keywords:** high-density lipoprotein (HDL) subclasses, paraoxonase-1 (PON1), small, dense, low-density lipoprotein (sdLDL)

## Abstract

The aim of this multicentric study was to assess the impacts of oxidative stress, inflammation, and the presence of small, dense, low-density lipoproteins (sdLDL) on the antioxidative function of high-density lipoprotein (HDL) subclasses and the distribution of paraoxonase-1 (PON1) activity within HDL in patients with ST-segment elevation acute myocardial infarction (STEMI). In 69 STEMI patients and 67 healthy control subjects, the lipoproteins’ subclasses were separated using polyacrylamide gradient (3–31%) gel electrophoresis. The relative proportion of sdLDL and each HDL subclass was evaluated by measuring the areas under the peaks of densitometric scans. The distribution of the relative proportion of PON1 activity within the HDL subclasses (pPON1 within HDL) was estimated using the zymogram method. The STEMI patients had significantly lower proportions of HDL2a and HDL3a subclasses (*p* = 0.001 and *p* < 0.001, respectively) and lower pPON1 within HDL3b (*p* = 0.006), as well as higher proportions of HDL3b and HDL3c subclasses (*p* = 0.013 and *p* < 0.001, respectively) and higher pPON1 within HDL2 than the controls. Independent positive associations between sdLDL and pPON1 within HDL3a and between malondialdehyde (MDA) and pPON1 within HDL2b were shown in the STEMI group. The increased oxidative stress and increased proportion of sdLDL in STEMI are closely related to the compromised antioxidative function of small HDL3 particles and the altered pPON1 within HDL.

## 1. Introduction

One of the clinical manifestations of atherosclerosis is acute myocardial infarction with two entities: ST-segment elevation acute myocardial infarction (STEMI) and non-ST-segment elevation acute myocardial infarction (NSTEMI). It is one of the most common causes of emergency admission and sudden death in developed countries and, in recent decades, in developing ones [1]. It has been shown that STEMI patients have a high in-hospital mortality and increased proinflammatory state, but more investigations are needed to determine the factors affecting the outcomes of these patients [2].

It is now accepted that dyslipidemia, inflammation, oxidative stress, and immune processes play important roles in the pathogenesis of atherosclerosis, but the mechanisms of their interactions are still unknown [3,4,5,6]. Although the optimal approach to cardiovascular risk management is based on lowering the concentration of low-density lipoprotein cholesterol (LDL-C), a significant percentage of patients with cardiovascular disease remain at risk of recurrent cardiovascular events [7]. One lipid biomarker with a great potential to increase this cardiovascular risk is small, dense, low-density lipoprotein (sdLDL), which is characterised by an enhanced atherogenicity because it is more susceptible to oxidative modification [8]. The first report on the presence of sdLDLs in patients with coronary artery disease was in 1983 [9]. Due to their small size and low density, sdLDLs pass more easily into the subendothelial space, where they are exposed to the harmful effects of oxidants, leading to the formation of oxidized low-density lipoproteins (oxLDLs). Once formed, oxLDL can stimulate LDL aggregation, the production of autoantibodies, and the formation of LDL-containing immune complexes [10].

On the other hand, high-density lipoprotein (HDL), well known for its antiatherosclerotic effects, constitutes a highly heterogeneous group of particles with different structures and functionalities [11,12,13]. It is considered that smaller HDL particles, in physiological conditions, have more potent atheroprotective properties compared to their larger counterparts [14]. However, it has been found that, in conditions associated with the progression of atherosclerosis, such as dyslipidemia, inflammation, and oxidative stress, HDL particles undergo structural changes that affect their atheroprotective capacity, making them dysfunctional [15,16]. Although HDL dysfunction and its relation to the changes in HDL subpopulations and antioxidative capacity in STEMI patients have been studied extensively, most studies report on alterations comparing large (HDL2) versus small (HDL3) subpopulations.

The crucial mechanism of atheroprotective HDL properties is reverse cholesterol transport, but the enzyme paraoxonase-1 (PON1) presented on HDL particles (PON1 within HDL) also plays a significant role in cardiovascular protection. The antioxidative role of PON1 is primarily reflected in its ability to hydrolyse lipid peroxides, thus protecting LDL particles and cell membranes from oxidation [17]. It is well known that PON1 is distributed across all HDL subclasses. Still, PON1 is considered to have the highest affinity for small HDL3 particles [18] and it has been shown that changes in the structure of these particles affect the PON1 activity [19,20]. However, it has not yet been clarified how altered HDL subclass compositions and distributions, as well as acute inflammation and oxidative stress, affect the distribution of the relative proportion of PON1 activity within the HDL subclasses (pPON1 within HDL) in patients with STEMI. Accordingly, we tested the hypothesis that the pPON1 within HDL particles differs concerning the HDL size and can be altered under the mentioned conditions. Therefore, the present study evaluates alterations in the proportions of the HDL subclasses and pPON1 within HDL, considering the increased proportion of sdLDL particles, inflammation, and oxidative stress among subjects with STEMI and healthy controls. According to our knowledge, this is the first study investigating the HDL2a, HDL2b, HDL3a, HDL3b, and HDL3c subclasses with the aim of providing more details about the alterations and relationships between different HDL particles and PON1 activity.

## 2. Results

Table 1 presents the demographic characteristics and laboratory parameters of the STEMI patients and healthy controls. A higher frequency of hypertension (HT) and smoking was identified in the STEMI patients. Moreover, the STEMI patients were older and had a lower body mass index (BMI) than the controls. Regarding the lipid status parameters, the triglycerides (TG) concentrations were higher and high-density lipoprotein cholesterol (HDL-C) concentrations were lower in the STEMI patients. Significantly higher concentrations of high sensitive C-reactive protein (hsCRP) were also found in the STEMI patients compared to the controls.

The difference in the HDL subclasses and pPON1 within the HDL proportions between the control and STEMI groups was evaluated using an analysis of covariance (ANCOVA), with age, BMI, HT, and smoking status included as covariates. The adjusted means for the HDL subclasses and pPON1 within the HDL proportions are shown in Table 2. The proportions of the HDL2, HDL2a, and HDL3a subclasses, as well as the pPON1 within HDL3b, were significantly lower, while the proportions of the HDL3, HDL3b, and HDL3c subclasses were higher in the STEMI patients compared to the control subjects.

Furthermore, we examined the proportions of the HDL subclasses (Table 3) and pPON1 within HDL (Table 4) concerning the presence and absence of STEMI and sdLDL proportions of > 50%.

The STEMI patients and controls with sdLDL proportions of > 50% had a higher proportion of HDL3a particles than their counterparts without increased sdLDL. Although no difference in the HDL3 subclasses between the STEMI patients and controls was observed, an sdLDL proportion of > 50% had a significant effect on the proportion of HDL3 particles. In particular, the HDL3 subclasses were more prevalent in individuals with sdLDL proportions of > 50%. The results are shown in Table 3.

**Figure 1:**
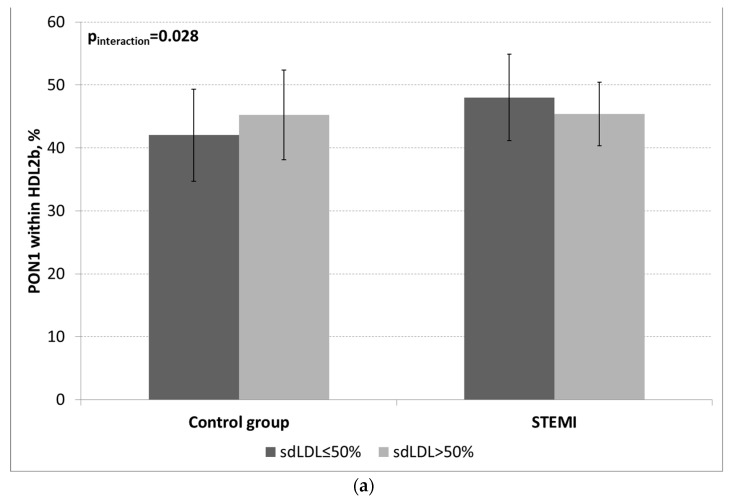

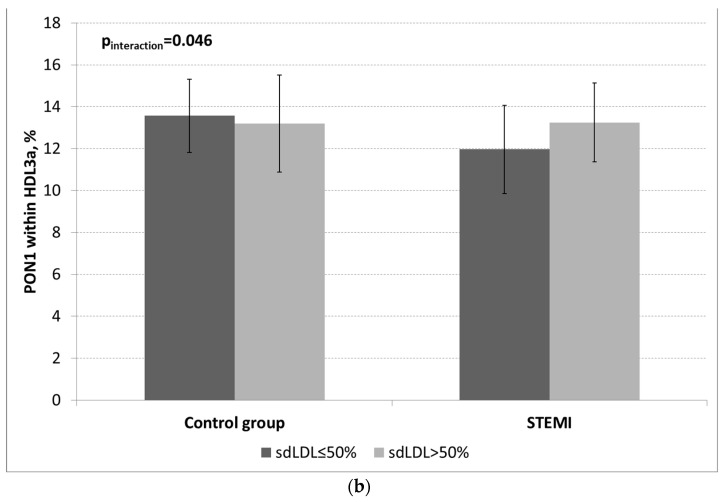
pPON1 within HDL2b and HDL3a regarding the prevalence of sdLDL higher or lower than 50% in STEMI and control subjects. (**a**)—mean pPON1 within HDL2b in STEMI and control subjects with a prevalence of sdLDL higher or lower than 50%; and (**b**)—mean pPON1 within HDL3a in STEMI and control subjects with a prevalence of sdLDL higher or lower than 50%.

The pPON1 within HDL2a showed a lower trend in subjects with an sdLDL proportion of > 50%, as shown in Table 4. However, STEMI status and sdLDL proportions of > 50% did not affect the pPON1 within the HDL2b and HDL3a subclasses, but there was a significant interaction between these two factors. The pPON1 within HDL2b was higher in the control subjects and lower in the STEMI patients with an increased proportion of sdLDL (Table 4 and Figure 1a) in comparison to their counterparts without increased sdLDL, contrary to the pPON1 within HDL3a, which was lower in the control group but higher in the STEMI patients (Table 4 and Figure 1b).

Table 5 shows the correlations between the lipid, inflammatory, and oxidative stress parameters for the PON1 within HDLs. In the control group, logsdLDL had a negative correlation with the PON1 within HDL2a, and in the patients, a correlation was shown between the logMDA and PON1 within HDL2b. The independent predictors of PON1 within HDLs in the STEMI patients and controls were investigated using a multiple linear regression analysis with forward selection. Investigated confounders are the concentrations of the logMDA, HDL, logTG, loghsCRP, and logsdLDL distributions. This analysis revealed that PON1 within HDL 3a in the STEMI group and PON1 within HDL 2a in the controls were significantly associated with the logsdLDL particle distribution, independent of the other investigated confounders. The logsdLDL particle distribution was inversely associated with PON1 within HDL 2a subclasses in the controls (−10.89 (−18.68–−3.09), *p* = 0.007) and positively with PON1 within HDL 3a (10.30 (0.34–20.29), *p* = 0.04) in the STEMI patients. On the other hand, higher logMDA concentrations are related to higher PON1 activity in the HDL2b subclasses (9.65 (1.64–17.66) *p* = 0.02), although only in STEMI patients. Multicollinearity was not detected; the tolerance values for all the domains were lower than 1.

## 3. Discussion

The current study demonstrates that STEMI is accompanied by modifications in the distribution of the HDL subclasses and changes in the functionality of HDL particles, reflected by alterations in the pPON1 within HDL. For the first time, our study analysed the distribution of pPON1 among the distinct HDL subclasses in STEMI patients.

Our study provided an intriguing finding, which was that elevated levels of total cholesterol (TC), LDL-C, and sdLDL were not observed in STEMI patients in comparison to healthy controls, as would be expected. The possible mechanisms for this phenomenon include the acute phase response associated with the up-regulation of LDL-C receptor activity [21], so the concentrations of these parameters are temporarily reduced. In addition, heterogeneity within the patient population can affect the observed differences in lipid parameters.

We found that our STEMI patients had increased proportions of small HDL3b and HDL3c subclasses and diminished pPON1 within HDL3. Therefore, the results of the present study indicate that the antioxidative ability of HDL3 particles in STEMI patients is reduced, and such findings can be explained by changes in the composition and maturation of HDL particles [20]. Bearing in mind that our STEMI patients had increased TG levels, it is reasonable to assume that TG accumulation in HDL particles, mediated by increased cholesterol ester transfer protein (CETP) activity, probably cause the reduced conformational stability of apo A-I and result in PON1 shedding from HDL, as described by Carnuta et al. [22] and Brites et al. [23]. These findings are in agreement with our previous research on patients with renal disease [24], as well as with the study of Nobecourt et al. [25], who demonstrated that patients with type 2 diabetes had diminished pPON1 within HDL3. Taken together, it could be suggested that the proatherogenic environment in various atherosclerosis-related diseases contributes to the reduced antioxidative properties of small HDL3 subclasses and the altered distribution of pPON1 within HDL3.

A high level of inflammation, which is present in STEMI, can affect HDL particles’ structures and functions, as well as PON1 activity. The increased hsCRP concentrations and decreased pPON1 within the HDL3 subclasses in our STEMI patients, in comparison to the control group, support the hypothesis that HDL subclasses’ changes during the intensive inflammatory period are associated with a decreased antioxidative capacity, as proposed by Bains et al. [26]. Additionally, Meisinger et al. [27] showed that, in subjects with high levels of inflammation, reflected by high concentrations of hsCRP and other inflammatory markers, their PON1 activity might be impaired, leading to dysfunctional HDL particles. Based on the above mentioned, alterations in pPON1 activity might result from HDL remodelling and the shifting PON1 between HDL particles during inflammation. Still, this process may also involve other factors, such as oxidative stress and dyslipidemia.

Regarding sdLDLs, it is generally expected that healthy subjects have a lower percentage of sdLDLs than cardiovascular disease (CVD) patients, but the results of the current study did not confirm this. There are several reasons for this finding: individual variations (genetic factors and lifestyle), as well as subclinical risk factors without signs of developed CVD (obesity, insulin resistance, or metabolic syndrome). All of these factors can contribute to differences in the presence of sdLDLs, which would require further investigation, including comprehensive lipid profiling with a subclass analysis and long- term follow-up to evaluate the actual risk associated with sdLDLs in a specific population.

Therefore, we further investigated the influence of STEMI and an increased sdLDL proportion on the distribution of the HDL subclasses and pPON1 within HDL. The obtained results showed that the STEMI patients and control subjects with increased sdLDLs had an increased proportion of HDL3 particles. In the control subjects, such a redistribution of HDL particles may be a protective response to the abundance of sdLDL particles. However, our STEMI patients were characterised by elevated TG and reduced HDL-C concentrations, with an abundance of small-sized HDL particles. In atherogenic dyslipidemia, increased CETP enhances HDL remodelling from large to small HDL subclasses and accelerates the catabolism of HDL. Namely, the transport of cholesteryl ester from cholesterol-rich lipoproteins to TG-rich lipoproteins is mediated by CETP, which results in the enrichment of HDL particles with TGs [12]. TG-enriched HDL particles are a substrate for hepatic lipase, leading to an increased accumulation of small HDL3 particles [14].

The analysis of pPON1 within the HDL subclasses showed that the control subjects with increased sdLDLs had higher pPON1 within HDL2b than their counterparts without increased sdLDLs. These data are consistent with the results of our previous study on women with polycystic ovary syndrome, which demonstrated a high proportion of PON1 activity within the HDL2b subclasses in women with LDL phenotype B [28]. However, the STEMI patients with increased sdLDLs had a lower pPON1 within HDL2b than their counterparts. This may be explained by the fact that some other factors, such as the products of oxidative stress and inflammation, affect the PON1 activity within HDL in STEMI. Indeed, a multivariable linear regression analysis revealed that higher concentrations of malondialdehyde (MDA), an important marker of lipid peroxidation, were independently associated with a higher pPON1 within HDL2b in the STEMI patients. This finding suggests that increased lipid peroxidation, rather than dyslipidemia per se, stimulated PON1 activity.

In contrast, the finding of increased pPON1 within HDL3a in the STEMI patients with an sdLDL proportion of >50%, in comparison to their counterparts with an sdLDL proportion of ≤50%, might be a consequence of the preponderance of the HDL3a subclasses in the STEMI group with increased sdLDLs. Furthermore, the multivariable regression analysis confirmed an independent association between the increased proportion of sdLDL and pPON1 within the HDL3a particles in the STEMI patients. Such association was not observed in the control subjects. Moreover, our finding that a higher pPON1 within HDL2a was inversely associated with sdLDL in the control group suggests that a preponderance of sdLDL particles diminished the PON1 within the HDL2a subclasses, even in the absence of cardiovascular disease. Since there is little known about the distribution of pPON1 within HDL subclasses, additional studies are needed to verify such presumptions.

Finally, some study limitations and strengths should be mentioned. The first limitation is undoubtedly the small number of study participants, as well as the need to determine some apolipoproteins (e.g., apo B), so further investigations with more subjects and more parameters should verify and expand our findings. Moreover, our research was conducted only on STEMI patients, so there is a need to include other groups of patients with CVD to more comprehensively assess the association between the HDL subclass distributions and pPON1 within HDL, especially in the presence of increased sdLDLs. On the other hand, our study’s importance is reflected in the fact that it provides additional insight into the mechanisms responsible for HDL dysfunction and provides data on the PON1 distribution within the distinct HDL subclasses in STEMI patients.

## 4. Materials and Methods

### 4.1. Subjects

This study, designed as a multicenter pilot study, included a total of 136 participants and was performed on STEMI patients, much like our recent previous study [29]. The study group consisted of 69 STEMI patients diagnosed according to the European Society of Cardiology guidelines [30], whose blood samples were collected at two Clinical Hospital Centres: “Bezanijska kosa” and “Zemun” in Belgrade. The control group consisted of 67 healthy persons who attended regular annual medical check-ups at a general hospital “Medigroup” in Belgrade and healthy volunteers employed at the Faculty of Pharmacy, University of Belgrade. The exclusion criteria from the study were malignancy, infection, endocrine, and hepatic diseases. Additionally, the control subjects underwent a detailed cardiology examination, including a stress test for coronary ischemia, an electrocardiogram, and a transthoracic echocardiography, to exclude cardiovascular disease, while the STEMI patients were not on lipid-lowering drugs.

Medical documentation was used to obtain data about the treatment and health status of the study participants. The essential data concerning each control subject included in the study, such as age, gender, weight, height, blood pressure, and smoking status, etc., were collected through interviews. In addition, the study protocol included a BMI calculation and evaluation of HT.

Every participant was informed about the study’s aims and provided written consent before entering the study. The whole research was planned and conducted following the principles of the Helsinki Declaration [31]. The study protocol was evaluated and approved by the Ethics Committees of the Faculty of Pharmacy, University of Belgrade, Clinical Hospital Centres: “Zemun” and “Bezanijska kosa” and General hospital “Medigroup”.

Plasma and serum were separated using centrifugation at 1500 rpm for 10 min and divided into multiple aliquots. The aliquots of each sample were stored at −80 °C until they were assayed. The samples were thawed immediately before the analyses.

### 4.2. Methods

The total cholesterol (TC), HDL-C, and TG were measured via routine enzymatic methods using a Dimension^®^ Xpand Plus biochemical analyser (Siemens, Munich, Germany). The Friedewald equation was used to calculate the LDL-C concentration [32]. The MDA concentration was determined using the method described by Girotti et al. [33], based on a spectrophotometric measurement of red-coloured MDA-thiobarbituric acid adduct at 535 nm. The immunoturbidimetric method (Tina-quant CRP, Roche, Indianapolis, USA) was used to obtain the concentration of hsCRP.

The LDL and HDL particles were separated using non-denaturing 3–31% polyacrylamide gradient gel electrophoresis, based on a modified version of the method described by Rainwater et al. [34]. The HDL and LDL particle size determination and subclass analyses were described in detail and published elsewhere [35]. The relative proportions of the HDL subclasses (in %) were determined by analysing the areas under the peaks that comprised the following ranges of HDL particle diameters: HDL2b (9.7–12.0 nm), HDL2a (8.8–9.7 nm), HDL3a (8.2–8.8 nm), HDL3b (7.8–8.2 nm), and HDL3c (7.2–7.8 nm). Accordingly, the HDL particles were grouped into large HDL2 (HDL2b and HDL2a) and small HDL3 (HDL3a, HDL3b, and HDL3c) subclasses. The relative proportion of sdLDL particles was assessed as the area of the densitometric scan at or below 25.5 nm.

After the electrophoretic separation of the HDL particles, we used the zymogram method described by Gugliucci et al. [36] to assess the pPON1 within HDL. The assay used phenylacetate as a substrate that hydrolysed phenol with the arylesterase activity of PON1. In the presence of K_3_[Fe(CN)_6_], the formed phenol reacted with 4-aminoantipyrine, creating a pink complex that correlated with the PON1 activity in a particular HDL subclass. The areas under the peaks of the densitometric scans of the samples reflected the pPON1 within HDL, as previously described [28].

The enrolled participants were divided into two groups according to their proportions of sdLDL and the impact of increased sdLDL on the distribution of the HDL subclasses and pPON1 within HDL. An increased proportion of sdLDL was considered in subjects with sdLDL of >50% [37].

### 4.3. Statistical Analysis

Normality assumptions were analysed using the Kolmogorov–Smirnov test. Student’s t-test was used to compare the variables with normal distributions. If the normality assumption was violated, the Mann–Whitney U test was used. Differences among the categorical variables were estimated using an χ^2^ test. ANCOVA was carried out to investigate the differences in the HDL subclasses and pPON1 within HDL between the patients and controls. An adjustment was performed for the demographic variables that were significantly different between the examined groups. A two-way analysis of variance (ANOVA) was used to evaluate the effects of an sdLDL proportion of > 50% in the STEMI group on the distribution of the HDL subclasses and pPON1 within HDL. The correlations were investigated using a Pearson correlation analysis. A multivariable linear regression analysis with forward selection was performed to examine the independent contributions of the lipid status, lipid peroxidation, and inflammatory parameters to the pPON1 within HDLs. To provide a linear relationship between the dependent and independent variables, we performed a logarithm transformation of the sdLDL, LDL, HDL, TG, hsCRP, and MDA values. To avoid multicollinearity, the VIF scores were below ten and a tolerance score above 0.2 was used.

The normally distributed continuous variables were presented as mean ± SD and skewed variables were expressed as median (low and high quartile) values, while the categorical variables were shown as relative frequencies. All the analyses were performed using PASW Statistics 27 (PASW Statistics for Windows, SPSS Inc., Chicago, IL, USA). All the tests were considered significant at *p* < 0.05 for the 2-tailed test.

## 5. Conclusions

Our data showed that STEMI patients are characterised by an impaired maturation and functionality of HDL particles and alterations in the distribution of pPON1 within HDL. Moreover, the antioxidative dysfunction of small HDL3 subclasses is intimately associated with oxidative stress and dyslipidemia in STEMI patients. Further research should investigate the potential underlying mechanisms for the observed changes in pPON1 within HDL related to other risk factors.

## Figures and Tables

**Table 1:** Demographics, clinical characteristics, and biochemical parameters in control subjects and STEMI patients.

Variable.	Control Group	STEMI Group	*p* Value
N	67	69	
Age, years	55 ± 9	61 ± 12	0.005
Prevalence of males, %	73.1	79.7	0.37
BMI, kg/m^2^	26.28 (24.18–29.50)	25.25 (23.37–27.70)	0.04
Smokers, %	19.4	58	<0.001
HT, %	38.8	61.8	0.001
TC, mmol/L	5.70 ± 1.09	5.54 ± 1.30	0.44
TG, mmol/L	1.40 (0.98–1.74)	1.70 (1.22–2.33)	0.007
HDL-C, mmol/L	1.34 (1.10–1.55)	0.95 (0.80–1.21)	<0.001
LDL-C, mmol/L	3.65 ± 1.06	3.67 ± 1.12	0.92
sdLDL, %	53.3 (43.0–58.5)	54.5 (48.1–58.7)	0.15
MDA, µmol/L	3.74 (3.26–4.28)	3.26 (2.44–6.11)	0.40
hsCRP, mg/L	0.7 (0.3–1.9)	3.7 (2.1–7.2)	<0.001

Normally distributed variables are presented as mean ± SD and compared by Student’s *t*-test, whereas non-normally distributed variables are presented as median (lower and upper quartile) and compared with Mann–Whitney U test. Categorical variables are expressed as absolute or relative frequencies and compared using the χ^2^ test. Abbreviations: BMI, body mass index; HDL-C, high-density lipoprotein cholesterol; hsCRP, high sensitive C-reactive protein; HT, hypertension; LDL-C, low-density lipoprotein cholesterol; MDA, malondialdehyde; sdLDL, small, dense, low-density lipoprotein; TC, total cholesterol; and TG, triglycerides.

**Table 2:** Distributions of HDL subclasses and PON1 within HDL in control subjects and STEMI patients.

Variable	Control Group	STEMI Group	*p* Value
N	67	69	
HDL2, %	62.1 (60.1–64.2)	56.6 (545–58.7)	<0.001
HDL2b, %	39.4 (37.5–41.3)	37.0 (35.0–38.9)	0.10
HDL2a, %	22.7 (21.7–23.7)	19.6 (18.7–20.6)	<0.001
HDL3, %	37.9 (35.8–39.9)	43.1 (41.1–45.1)	<0.001
HDL3a, %	18.6 (17.7–19.5)	17.0 (16.1–17.9)	<0.03
HDL3b, %	9.5 (8.9–10.2)	11.6 (10.8–12.3)	0.001
HDL3c, %	9.8 (9.5–11.0)	14.6 (13.3–15.8)	<0.001
pPON1 within HDL2, %	65.4 (63.4–67.3)	11.3 (9.8–12.9)	0.09
pPON1 within HDL2b, %	43.6 (41.7–44.4)	46.4 (44.4–48.4)	0.06
pPON1 within HDL2a, %	21.8 (20.5–23.1)	21.7 (20.3–23.0)	0.87
pPON1 within HDL3, %	34.6 (32.7–36.6)	31.8 (29.9–34.1)	0.09
pPON1 within HDL3a, %	13.6 (13.0–14.1)	12.7 (12.1–13.3)	0.07
pPON1 within HDL3b, %	9.0 (8.5–9.6)	7.9 ± 2.1	0.006
pPON1 within HDL3c, %	12.5 ± 5.9	10.9 ± 3.7	0.12

Data are presented as adjusted means and 95% confidence intervals for means and compared with ANCOVA analyses. Means are adjusted for age, BMI, HT, and smoking status. Abbreviations: HDL, high-density lipoprotein; and pPON1 within HDL, the relative proportion of paraoxonase-1 (PON1) activity within high-density lipoprotein (HDL) subclasses.

**Table 3:** HDL subclasses distribution concerning the presence of disease and sdLDL proportion > 50%.

Variable	sdLDL Proportion	Control Group	STEMI Group	*p* (sdLDL Groups)	*p* (STEMI vs. Control)	*p* (Interaction)
HDL2, %	sdLDL ≤ 50%	63.4 ± 7.4	59.4 ± 9.0	0.04	0.15	0.24
	sdLDL > 50%	58.5 ± 9.7	58.0 ± 6.8			
HDL2b, %	sdLDL ≤ 50%	40.6 ± 5.9	38.3 ± 8.7	0.15	0.85	0.15
	sdLDL > 50%	36.6 ± 9.3	38.3 ± 5.8			
HDL2a, %	sdLDL ≤ 50%	22.8 ± 3.9	21.1 ± 3.0	0.08	0.004	0.76
	sdLDL > 50%	31.8 ± 3.4	19.7 ± 3.9			
HDL3, %	sdLDL ≤ 50%	36.4 ± 6.9	40.6 ± 9.0	0.04	0.15	0.14
	sdLDL > 50%	41.5 ± 9.7	41.6 ± 6.5			
HDL3a, %	sdLDL ≤ 50%	17.8 ± 3.3	16.0 ± 2.8	0.03	<0.001	0.30
	sdLDL > 50%	19.8 ± 4.1	16.7 ± 2.9			
HDL3b, %	sdLDL ≤ 50%	9.0 ± 2.6	10.8 ± 2.8	0.06	0.01	0.36
	sdLDL > 50%	10.5 ± 3.2	11.3 ± 2.2			
HDL3c, %	sdLDL ≤ 50%	9.5 ± 6.0	13.8 ± 5.3	0.37	<0.001	0.29
	sdLDL > 50%	11.3 ± 4.4	13.7 ± 4.5			

Data are expressed as mean ± SD and compared using two-way ANOVA; HDL subclasses are expressed as relative proportions estimated by measuring the areas under the peaks of densitometric scans. Note: number of control subjects with the proportion of sdLDL < 50% was N = 27 and sdLDL > 50% was N = 40; and number of STEMI patients with the proportion of sdLDL < 50% was N = 20 and sdLDL > 50% was N = 49. Abbreviations: HDL, high-density lipoprotein; and sdLDL, small, dense, low-density lipoprotein.

**Table 4:** Proportions of PON1 within HDLs concerning the presence of disease and sdLDL proportion > 50%.

Variable	sdLDL Proportion	Control Group	STEMI Group	*p* (sdLDL Groups)	*p* (STEMI vs. Control)	*p* (Interaction)
pPON1 within HDL2b, %	sdLDL ≤ 50%	42.0 ± 7.3	48.0 ± 6.8	0.82	0.09	0.03
	sdLDL > 50%	45.3 ± 7.1	45.4 ± 5.0			
pPON1 within HDL2a, %	sdLDL ≤ 50%	22.9 ± 5.5	22.7 ± 3.9	0.04	0.45	0.30
	sdLDL > 50%	20-2 ± 3.1	21.8 ± 4.9			
pPON1 within HDL3, %	sdLDL ≤ 50%	35.1 ± 7.0	29.3 ± 6.7	0.28	0.02	0.14
	sdLDL > 50%	34.5 ± 7.8	32.8 ± 5.5			
pPON1 within HDL3a, %	sdLDL ≤ 50%	13.6 ± 1.7	11.9 ± 2.1	0.27	0.06	0.046
	sdLDL > 50%	13.2 ± 2.3	13.2 ± 1.9			
pPON1within HDL3b, %	sdLDL ≤ 50%	9.3 ± 1.9	7.6 ± 1.9	0.89	0.003	0.15
	sdLDL > 50%	8.7 ± 1.8	8.1 ± 2.1			
pPON1 within HDL3c, %	sdLDL ≤ 50%	12.2 ± 4.3	9.8 ± 3.4	0.29	0.08	0.53
	sdLDL > 50%	12.6 ± 6.7	11.5 ± 3.8			

Data are expressed as mean ± SD and compared with two-way ANOVA; pPON1 within different HDL subclasses is expressed as relative proportion estimated by measuring the areas under the peaks of densitometric scans. Note: number of control subjects with the proportion of sdLDL < 50% was N = 27 and sdLDL > 50% was N = 40; and number of STEMI patients with the proportion of sdLDL < 50% was N = 20 and sdLDL > 50% was N = 49. Abbreviations: pPON1 within HDL, the relative proportion of paraoxonase-1 (PON1) activity within high-density lipoprotein (HDL) subclasses; and sdLDL, small dense low-density lipoprotein.

**Table 5:** Correlation of lipid, inflammatory, and oxidative stress parameters with PON1 within HDLs.

**Controls** **r**					
	**PON1 within** **HDL2b**	**PON1 within** **HDL2a**	**PON1 within** **HDL3a**	**PON1 within** **HDL3b**	**PON1 within** **HDL3c**
HDL	0.054	0.121	−0.098	−0.027	−0.113
logTG	−0.017	−0.199	0.050	0.110	0.115
logsdLDL	0.070	−0.375 **	−0.038	0.054	0.187
logCRP	0.204	−0.044	−0.109	−0.0117	−0.132
logMDA	0.086	−0.132	0.089	0.069	−0.063
**STEMI Patients** **r**					
	**PON1 within** **HDL2b**	**PON1 within** **HDL2a**	**PON1 within** **HDL3a**	**PON1 within** **HDL3b**	**PON1 within** **HDL3c**
HDL	0.021	0.002	0.117	−0.129	0.035
logTG	0.066	−0.208	−0.013	0.046	0.153
logsdLDL	0.029	−0.129	0.177	−0.053	0.059
logCRP	−0.199	0.039	0.248	0.096	0.053
logMDA	0.342 *	−0.133	−0.125	−0.106	−0.196

* *p* < 0.05, ** *p* < 0.01, r-Pearson correlation coefficient TG, LDL, CRP, and MDA values were logarithmically transformed before analysis. Abbreviations: MDA, malondialdehyde; pPON1 within HDL, the relative proportion of paraoxonase-1 (PON1) activity within high-density lipoprotein (HDL) subclasses; and sdLDL, small, dense, low-density lipoprotein.

## Data Availability

The data in this study could be made available upon reasonable request to the corresponding author.
